# Inhalation of 1-1-difluoroethane: A Rare Cause of Pneumopericardium

**DOI:** 10.7759/cureus.3503

**Published:** 2018-10-27

**Authors:** Erika L Faircloth, Jose Soriano, Deep Phachu

**Affiliations:** 1 Internal Medicine, University of Connecticut, Farmington, USA; 2 Internal Medicine, Saint Francis Hosptial and Medical Center, Hartford, USA

**Keywords:** 1-1-difluoroethane, fluorinated hydrocarbon, cardiology, pneumopericardium, pneumomediastinum, inhalant abuse

## Abstract

We report a case of a 32-year-old man with a past medical history of ethanol use disorder who was brought in unresponsive after inhaling six to 10 cans of the computer cleaning product, Dust-Off. After regaining consciousness, he endorsed severe, pleuritic chest and anterior neck pain. Labs were notable for elevated cardiac enzymes, acute kidney injury, and his initial electrocardiogram (ECG) revealed a partial right bundle branch block with a prolonged corrected QT interval (QTc). On chest X-ray as well as chest computed tomography, the patient was found to have pneumomediastinum, pneumopericardium, and subcutaneous emphysema. The patient’s course was uneventful and he was discharged home two days later after extensive substance abuse cessation counseling. Intentionally inhaling toxic substances, also known as “huffing,” is a dangerous new trend with significant consequences that clinicians need to be aware of and suspect in young patients presenting with chest pain. We present a rare case of pneumopericardium induced by inhalation of Dust-Off (1-1-difluoroethane).

## Introduction

1-1-difluoroethane is a colorless, odorless gas [1]. Intentional inhalation of fluorinated hydrocarbons, which are present in computer cleaning products such as Dust-Off, is a worrisome trend with severe consequences. The National Survey on Drug Use and Health’s Monitoring the Future survey in 2016 found that 9.1% of people 12 years and older have used inhalants in their lifetime [2]. Other reports note that 11% of high school students have experimented with an inhalant at least once [3]. The sought after euphoria or “high” can also be accompanied by central nervous system depression due to the extreme lipophilic properties of the gas and increased gamma-aminobutyric acid type A_ _receptor affinity [1,3]. Inhalation can cause hypoxemia by displacement of oxygen and can lead to kidney injury as a result of byproduct degradation. Frostbite can occur due to skin freezing [1]. Cardiac consequences are severe and vast. Direct injury can lead to cardiomyopathy. In addition, fluorinated hydrocarbons are myocardial sensitizers and can predispose patients to cardiac dysrhythmias by catecholamine surges, and alteration of the potassium current. Atrial and atrioventricular node conduction time prolongation occur secondary to alteration in calcium liberation from the sarcoplasm reticulum [1].

## Case presentation

**Figure 1:**
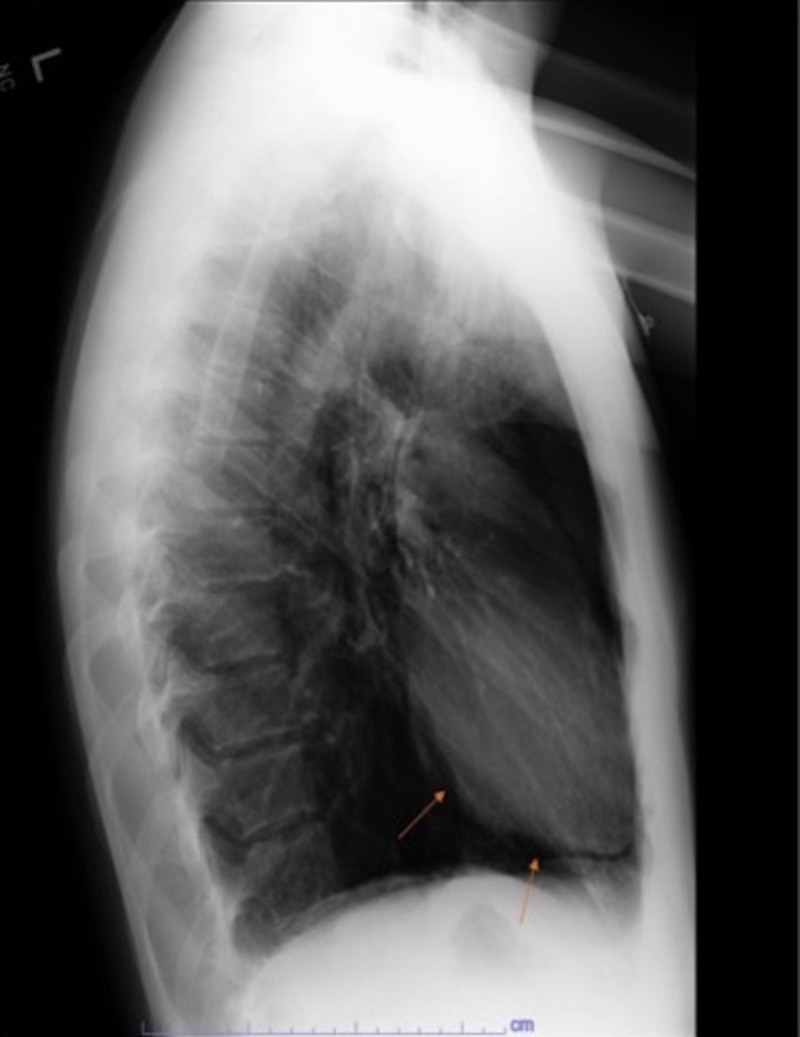
Lateral chest X-ray demonstrating pneumopericardium.

A 32-year-old homeless man with a past medical history of ethanol use disorder (sober for 40 days prior to presentation) was brought in by emergency medical personnel after being found unresponsive in a Walmart parking-lot. After regaining consciousness, the patient stated that the last thing he remembered was “huffing” six to ten cans of the computer cleaning product, Dust-Off. He endorsed sharp, substernal chest and anterior neck discomfort made worse by deep inspiration. Initially, he was given two amps of bicarbonate, as well as empiric antibiotics. Physical exam was unremarkable with the exception of what appeared to be frost-bite on his fingertips of the right hand. Laboratory work was notable for leukocytosis of 17.7 thousand per microliter (K/uL), sodium of 127 millimoles per liter (mmol/L), potassium of 3.5 mmol/L, chloride of 90 mmol/L, bicarbonate of 20 mmol/L, creatinine of 1.63 milligram per deciliter (mg/dl), normal liver enzymes and a negative toxicology screen. Troponin was elevated to 4.34 nanograms per milliliter (ng/mL), creatinine kinase was 1163 units per liters (U/L) and creatinine kinase-muscle/brain (CK-MB) was 21.3 ng/mL. His initial electrocardiogram revealed sinus rhythm with a partial right bundle branch block and a prolonged corrected QT segment (QTc) of 518 milliseconds. On chest X-ray, the patient was found to have pneumomediastinum and pneumopericardium (Figure 1).

**Figure 2:**
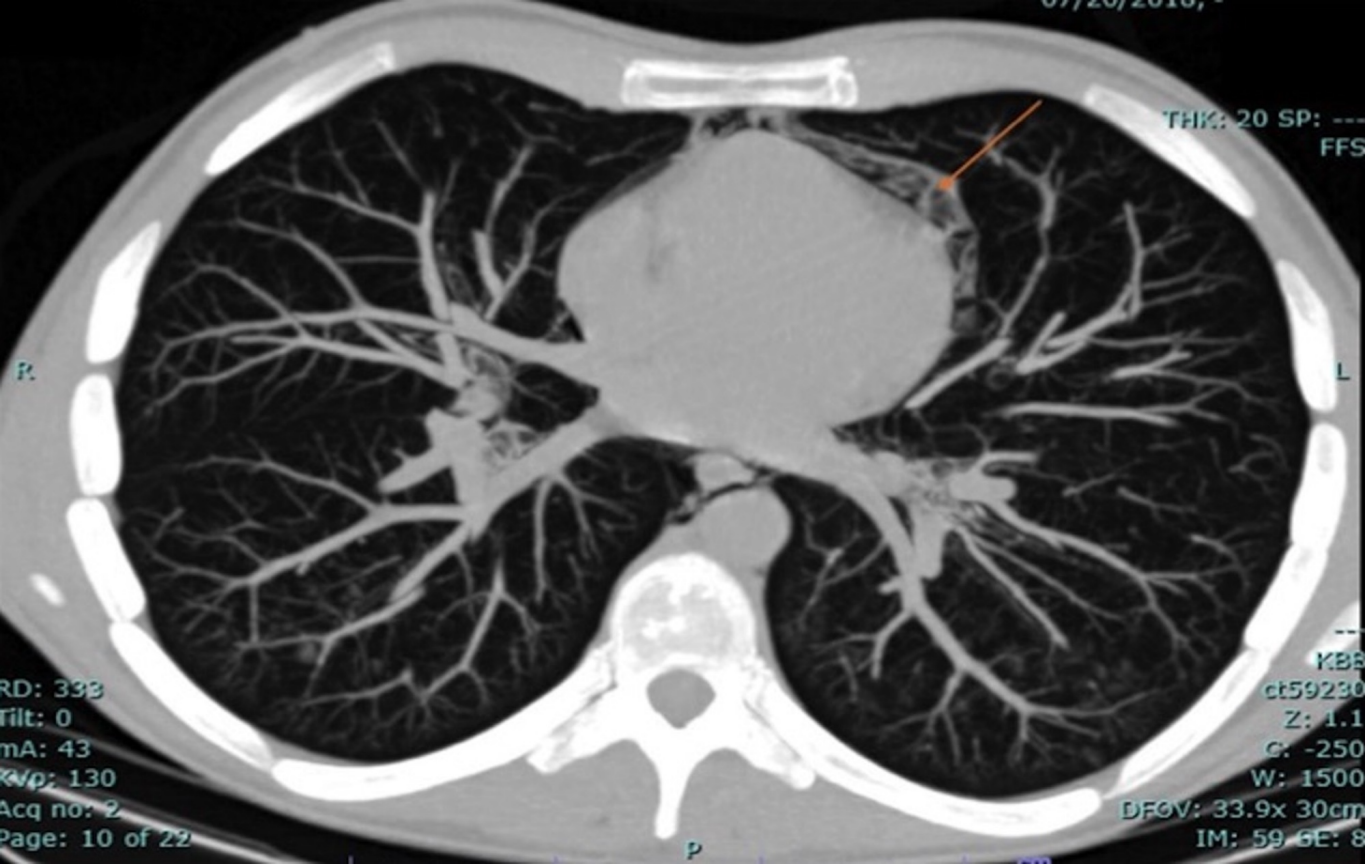
Computed tomography of the chest demonstrating pneumopericardium.

Computed tomography of the chest demonstrated extensive air around the tracheal and laryngeal structures, extending down the cervical tissue planes and into the mediastinum and pericardium (Figure 2).

Cardiothoracic surgery was consulted and the decision was made to observe the patient as he remained hemodynamically stable and he was protecting his airway. The patient did well, and prior to discharge, his kidney function improved, he had a normal echocardiogram, normalization of his electrocardiogram, and minimal residual mediastinal and pericardial air on repeat chest X-ray. He was provided extensive education and support for cessation of inhalant and alcohol use.

## Discussion

There have been many case reports about the dangers of abusing 1-1-difluoroethane including cardiomyopathy, myocarditis, acute kidney injury, and skeletal fluorosis [3-6]. To our knowledge, this is the first reported case of pneumopericardium secondary to huffing fluorinated hydrocarbons. Pneumopericardium itself is a rare entity often caused by barotrauma leading to alveolar rupture. Alveolar rupture allows air to escape interstitially around the bronchi into the mediastinum. The air can further rupture into the pleural space causing a pneumothorax, can tract up through the subcutaneous planes resulting in subcutaneous emphysema, or can get into the pericardium resulting in pneumopericardium [7]. Barotrauma is most frequently seen in mechanically ventilated patients with high fractions of inspired air, high peak airway pressures, large tidal volumes, and high-end expiratory pressures. It can also be seen in divers, blunt trauma and fistula formation [7]. Pneumopericardium can be due to tension or can be spontaneous, which is more commonly seen secondary to barotrauma [8]. Cummings et al. reviewed 252 patients with pneumopericardium and found that 37% developed cardiac tamponade, most frequently in blunt trauma cases or neonates on positive-pressure ventilation. The outcomes of 221 of the 252 patients was reported: 57% of those with tension pneumopericardium died, and 56% of those with spontaneous pneumopericardium died illustrating the high morbidity of inhalants [9].

## Conclusions

In this report, we document a rare case of pneumopericardium induced by intentionally inhaling the computer cleaning product, Dust-Off. Many people who “huff” are not as lucky as our patient who was discharged symptom-free. Pneumopericardium can be a dangerous entity and it should not be taken lightly. As clinicians, we need to screen patients for substance abuse regularly and educate our patients on the real, and potentially deadly, risks of household items used improperly.
